# Targeting PFKFB3 to enhance CDK4/6 inhibitor response in ER+ breast cancer

**DOI:** 10.21203/rs.3.rs-8412774/v1

**Published:** 2026-02-10

**Authors:** Sucheta Telang, Brian F Clem, Ariamna A Herrera Miret, Leanne Price, Susan M Dougherty, Anna Schmitz, Xinmin Yin, Xipeng Ma, Xiang Zhang, Jason Chesney, Yoannis Imbert-Fernandez

**Affiliations:** University of Louisville; University of Louisville; University of California; University of Louisville; University of Louisville; University of Dayton; University of Louisville; University of Louisville; University of Louisville; University of Louisville; University of Louisville

**Keywords:** CDK4/6 inhibitor, estrogen receptor, breast cancer, PFKFB3, glucose metabolism

## Abstract

**Background:**

Cyclin-dependent kinase 4 and 6 (CDK4/6) inhibitors are widely used in the treatment of estrogen receptor–positive (ER^+^) breast cancer; however, the metabolic adaptations induced by CDK4/6 inhibition remain incompletely defined. In ER^+^ breast cancer, estrogen signaling plays a central role in coordinating cell cycle progression and metabolic programs that support tumor growth. Glycolytic flux is regulated at the level of phosphofructokinase-1 (PFK1) through the inducible enzyme 6-phosphofructo-2-kinase/fructose-2,6-bisphosphatase 3 (PFKFB3), which is transcriptionally regulated by estrogen receptor signaling and has been shown to promote glycolysis and proliferation in ER^+^ breast cancer cells. Yet, how CDK4/6 inhibition intersects with estrogen-regulated glycolytic control to rewire glucose utilization in ER^+^ breast cancer has not been explored.

**Methods:**

Glucose metabolism was assessed using extracellular flux analysis, untargeted metabolomics, and stable isotope tracing with uniformly labeled ^13^C-glucose in ER + breast cancer cell lines. *In vivo* metabolic tracing was performed following bolus administration of [U-^13^C]-glucose. The effects of pharmacologic PFKFB3 inhibition, alone and in combination with CDK4/6 inhibitors, were evaluated *in vitro* and in patient-derived xenograft (PDX) models. Statistical analyses were performed using appropriate tests with correction for multiple comparisons where applicable.

**Results:**

CDK4/6 inhibition increased glycolytic flux, as evidenced by elevated basal and compensatory glycolysis, accumulation of early glycolytic intermediates, and increased ^13^C labeling of fructose 1,6-bisphosphate. PFKFB3 silencing abrogated the CDK4/6 inhibitor-induced increase in glycolytic flux. Despite increased glycolysis, stable isotope tracing revealed markedly reduced incorporation of glucose-derived carbon into nucleotide biosynthesis and lipid-associated metabolites, consistent with reduced anabolic demand during G1 cell cycle arrest. *In vivo* glucose tracing demonstrated a dissociation between increased glycolytic flux and downstream biosynthetic utilization. Pharmacologic inhibition of PFKFB3 imposed additional constrains on glucose utilization and significantly enhanced the antitumor efficacy of CDK4/6 inhibition in PDX models.

**Conclusions:**

CDK4/6 inhibition rewires glucose metabolism in ER + breast cancer by increasing glycolytic flux while limiting downstream glucose utilization, resulting in heightened reliance on regulated glycolytic control to maintain metabolic homeostasis during cell cycle arrest. Disruption of this adaptive metabolic state through PFKFB3 inhibition enhances the antitumor effects of CDK4/6 inhibition and supports the therapeutic potential of targeting glycolytic regulation in combination with CDK4/6 inhibitor-directed therapies.

## Background

Estrogen receptor alpha (ERα) drives the progression of ER+/HER2-negative breast cancer and is the molecular basis for current therapeutic approaches(1–3). Endocrine therapy has steadily improved patient outcomes, evolving from selective estrogen receptor modulators (SERMs), to aromatase inhibitors (AIs), and most recently to selective estrogen receptor degraders (SERDs)(2, 4). A major therapeutic breakthrough has been the addition of CDK4/6 inhibitors (palbociclib, ribociclib, abemaciclib) to endocrine therapy, which now represents the standard of care for advanced ER + breast cancer(5–7). Compared with endocrine therapy alone, CDK4/6 inhibitor combinations nearly double progression-free survival and significantly improve overall survival (8–10).

Mechanistically, CDK4/6 inhibitors suppress CDK4/6-mediated phosphorylation of the retinoblastoma protein (Rb) at Ser 780, thereby maintaining Rb in its active state and inducing G1 cell cycle arrest with consequent inhibition of tumor proliferation(11, 12). Despite their clinical impact, the cellular programs engaged downstream of Rb activation in response to CDK4/6 inhibition remain incompletely defined. In particular, the mechanisms that determine response, adaptive tumor persistence, and eventual resistance are not fully understood, underscoring the need to identify pathways downstream of CDK4/6 that contribute to therapeutic sensitivity.

Emerging evidence indicates that CDK4/6 inhibitors reprogram cancer cell metabolism, altering glucose utilization, mitochondrial activity, and nucleotide biosynthesis(13–18). Glycolysis, in particular, has been implicated as a pathway that shapes the response to CDK4/6 inhibition, and inhibition of glucose metabolism has been found to enhance palbociclib efficacy in preclinical models (19). These findings suggest that metabolic plasticity may enable tumor cell maintenance during treatment, thereby limiting the depth and/or durability of CDK4/6 inhibitor responses.

Among glycolytic regulators, 6-phosphofructo-2-kinase/fructose-2,6-bisphosphatase 3 (PFKFB3) is a key rate-controlling enzyme that governs the synthesis of fructose-2,6-bisphosphate (F2,6BP), a potent allosteric activator of phosphofructokinase 1 (PFK-1)(20, 21). We previously demonstrated that ER promotes PFKFB3 expression and that PFKFB3 is required for glucose metabolism and proliferative capacity in ER + breast cancer cells(22). However, whether CDK4/6 inhibitors engage PFKFB3-dependent metabolic programs, and whether targeting this metabolic node can enhance therapeutic efficacy of CDK4/6 inhibition remains unknown.

Here, we demonstrate that CDK4/6 inhibitors increase PFKFB3 expression and activity in ER + breast cancer cells, leading to enhanced glycolytic metabolism. Pharmacological inhibition of PFKFB3 with the selective small-molecule inhibitor PFK-158 disrupts this adaptive metabolic response and potentiates the antitumor effects of CDK4/6 blockade *in vitro* and *in vivo*. Together, these findings identify PFKFB3 as a critical mediator of adaptive metabolic remodeling following CDK4/6 inhibition that can be therapeutically leveraged to enhance treatment efficacy in ER + breast cancer.

## Methods

### Cell culture

MCF7 (HTB-22) and T47D (HTB-133) human breast cancer cell lines were obtained from the American Type Culture Collection (ATCC) and maintained at 37°C with 5% CO_2_. MCF7 cells were cultured in Improved Minimum Essential Medium (IMEM; Corning) supplemented with 10% fetal bovine serum (FBS; Invitrogen). T47D cells were cultured in RPMI-1640 (Gibco) supplemented with 10% FBS (Invitrogen) and human recombinant insulin (Lonza). Cell lines were verified by short tandem repeat (STR) profiling, and all experiments were performed using cells within 15 passages after reconstitution from frozen stocks.

#### Drug Treatments

Palbociclib (S1116; Selleckchem), ribociclib (S7440; Selleckchem), and PFK-158 (HY-12203; MedChem Express) were used in this study. Palbociclib stock solutions were prepared in sterile water, whereas ribociclib and PFK-158 stock solutions were dissolved in dimethyl sulfoxide (DMSO). All compounds were diluted in complete culture medium to the indicated final concentrations immediately prior to treatment. Vehicle controls (water or DMSO, as appropriate) were included in all experiments.

#### RNA extraction and Real Time PCR

Cells were seeded in six-well plates at a density of 1.5 × 10^5^ cells per well and allowed to adhere overnight prior to treatment. Cells were then treated with the indicated drug concentrations for the specified durations. Total RNA was isolated using the RNeasy Mini Kit (Qiagen) according to the manufacturer’s instructions. Quantitative real-time PCR (qRT–PCR) was performed using TaqMan Gene Expression Assays (Applied Biosystems). PFKFB3 expression was assessed using a validated human TaqMan probe (Hs00190079), with β-actin used as the endogenous control (Hs01060665_g1). Relative gene expression was calculated using the comparative C_T_ (ΔΔC_T_) method as previously described (22) and normalized to the corresponding vehicle controls. Because two independent vehicle control samples were included for each experiment, data were not normalized to a fixed value of 1, allowing for statistical comparison across treatment groups. Statistical significance was determined using two-way analysis of variance (ANOVA). Data represent three independent experiments; each performed with two biological replicates.

#### Western blot analyses

Western blot analyses

Cells were harvested and lysed in RIPA buffer supplemented with protease and phosphatase inhibitors (PhosSTOP; Roche) as previously described (22). Cell lysates were cleared by centrifugation, and protein concentrations were determined using the Pierce BCA Protein Assay Kit (Thermo Fisher Scientific, Waltham, MA, USA). Equal amounts of protein were resolved on 10–12% SDS–polyacrylamide gels and transferred onto Hybond-PVDF membranes (GE Healthcare Life Sciences, Pittsburgh, PA, USA).

Membranes were blocked and incubated overnight at 4°C with the following primary antibodies: phospho-PFKFB3 (Ser461) (PA5-114619; Invitrogen), PFKFB3 (13763-1-AP; Proteintech), GLUT1 (sc-377228; Santa Cruz Biotechnology), hexokinase II (2867; Cell Signaling Technology), PFK1 (8164; Cell Signaling Technology), pyruvate dehydrogenase (PDH; 3205; Cell Signaling Technology), lactate dehydrogenase A (LDHA; 3582; Cell Signaling Technology), and β-actin (A5316; Sigma-Aldrich). Membranes were then incubated with species-specific horseradish peroxidase–conjugated secondary antibodies (anti-mouse or anti-rabbit IgG; Thermo Fisher Scientific, Waltham, MA, USA). Protein bands were visualized using enhanced chemiluminescence (ECL) reagents according to the manufacturer’s instructions (GE Healthcare Life Sciences, Pittsburgh, PA, USA). Scanned band intensities were quantified using UN-SCAN-IT Gel 6.3 software (Silk Scientific, Orem, UT, USA). Densitometric values were normalized as indicated. Quantified values are expressed relative to control.

#### Glucose Uptake

MCF7 and T47D cells were seeded in six-well plates at a density of 2 × 10^6^ cells per well and treated with vehicle or the indicated concentrations of palbociclib or ribociclib for 48 hours. Following treatment, cells were washed twice with pre-warmed glucose- and pyruvate-free DMEM (DMEM − Glc/−Pyr/+Gln) supplemented with 10% dialyzed fetal bovine serum and incubated in the same medium for 30 minutes. Cells were then incubated with 2-[^14^C]-deoxy-D-glucose (5 μL; NEC495A050UC, Revvity, Boston, MA, USA) for 15 minutes at 37°C in 5% CO_2_. After incubation, cells were washed three times with ice-cold glucosefree RPMI medium (− Glc/−Pyr/+Gln), lysed in 0.1% SDS, and lysates were transferred to scintillation vials containing Ultima Gold scintillation fluid (6013321, Revvity, Waltham, MA, USA). Radioactivity was measured using a Tri-Carb 2910 TR liquid scintillation analyzer according to the QuantaSmart^™^ reference manual.

Because CDK4/6 inhibitor–treated cells undergo G1 cell cycle arrest while continuing to increase in cell size, normalization to metabolic endpoints that scale with cell mass (e.g., protein content or MTT) can confound interpretation of metabolic flux measurements(23). Therefore, glucose uptake was normalized to cell number. For normalization, parallel plates were seeded and treated identically, and cell numbers were determined at the time of harvest using a hemocytometer.

### Glycolytic Rate Assay

Cells were seeded in Seahorse XF24 cell culture microplates (Agilent Technologies) at a density of 2.0 × 10^4^ cells per well and allowed to adhere overnight. Cells were then treated with vehicle or the indicated drug conditions for 48 hours in complete growth medium. Following treatment, extracellular flux measurements were performed using the Seahorse XF Glycolytic Rate Assay Kit (103344-100; Agilent Technologies, Santa Clara, CA, USA) according to the manufacturer’s instructions and analyzed on an Agilent Seahorse XFe24 Analyzer. Glycolytic rate parameters were calculated using Agilent Seahorse Analytics XF software.

Following completion of the glycolytic rate assay, cell number was determined from the same Seahorse assay wells using the FluoroReporter^®^ Assay (Invitrogen). Cell counts were calculated by interpolation from a standard curve generated under identical assay conditions. Seahorse metabolic flux data were normalized to cell number to account for CDK4/6 inhibitor–induced cell cycle arrest and associated changes in cell size.

#### [U-C]-glucose tracer studies

MCF7 cells were seeded in 10-cm dishes (#430167, Corning,) at a density of 3 × 10^6^ cells/dish and treated with vehicle control or 500 nM palbociclib for 24 hours. Following this initial treatment period, culture medium was replaced with glucose- and pyruvate-free DMEM supplemented with 1 g/L [U-^13^C]glucose (Cambridge Isotope Laboratories), 10% dialyzed fetal bovine serum, and the corresponding drug treatments and incubated for an additional 24 hours. At the end of the labeling period, cells were washed three times with ice-cold 1× phosphate-buffered saline (PBS), and metabolism was rapidly quenched with cold acetonitrile. Metabolites were extracted using an acetonitrile:water:chloroform mixture (2 mL:1 mL:740 μL). Samples were centrifuged at 3,000 × g for 20 minutes at 4°C to separate polar, lipid, and cellular debris layers. The remaining cell debris was subjected to a second extraction using chloroform:methanol supplemented with butylated hydroxytoluene (2:1, 1 mM) and centrifuged at 22,000 × g for 20 minutes at 4°C. Polar and lipid fractions from both extraction steps were pooled separately. The polar fraction was vacuum-dried by lyophilization and reconstituted in 100 μL of 50% acetonitrile, followed by vigorous vortexing for 3 minutes. Samples were centrifuged at 14,000 rpm for 20 minutes at 4°C, and 80 μL of the clarified supernatant was collected for two-dimensional liquid chromatography–tandem mass spectrometry (2DLC–MS/MS) analysis. Extracted metabolites were subsequently lyophilized and analyzed by Liquid Chromatography Mass Spectrometry (LC/MS) at the Center for Regulatory and Environmental Analytical Metabolomics (CREAM) core facility at the University of Louisville as previously described (16, 24). Relative abundance levels were calculated by the sum of all isotopologue intensities of the specified metabolite divided by cell number.

#### CRISPR/Cas9-Mediated PFKFB3 Knockout

CRISPR/Cas9-Mediated PFKFB3 Knockout

PFKFB3 knockout (PFKFB3-KO) cell lines were generated using Dharmacon^™^ Edit-R^™^ all-in-one lentiviral CRISPR–Cas9 sgRNA constructs (Horizon Inspired Cell Solutions). Two independent sgRNAs targeting human PFKFB3 were used (clone IDs VSGHSOH_37741350 and VSGHSOH_37741355; Cat. No. VSGH12682–16EG5209). A non-targeting sgRNA lentiviral construct (VSGC11964) was used as a control. Cells were seeded in 24-well plates at a density of 1 × 10^5^ cells and transduced at approximately 60% confluency using a multiplicity of infection (MOI) of 0.3. Six hours after transduction, viral supernatant was replaced with complete growth medium. Twenty-four hours later, cells were placed under puromycin selection to enrich for successfully transduced cells. Puromycin-resistant populations were expanded and subsequently passaged for downstream analyses.

### Synergy studies

For drug interaction studies, cells were seeded in 96-well plates at a density of 5,000 cells per well and allowed to adhere overnight. Cells were treated with increasing concentrations of CDK4/6 inhibitors alone or in combination with PFKFB3 knockout using a matrix-based experimental design. Drug concentration ranges were centered around the half-maximal inhibitory concentration (IC_50_) for each drug and cell line, with concentrations titrated above and below the IC_50_ to generate full dose–response matrices. Cell number was determined using the FluoroReporter^®^ Assay (Invitrogen), with counts interpolated from a standard curve generated under identical assay conditions. Combination data was formatted as concentration–response matrices. Drug interaction analyses were performed using the SynergyFinder web application (SynergyFinder 2.0), and synergy scores were calculated using the Highest Single Agent (HSA) reference model(25). Combination effects were interpreted based on SynergyFinder-derived HSA synergy scores.

### Mouse studies

All animal experiments were performed in compliance with practices described in the National Institutes of Health Guide for the Care and Use of Laboratory Animals and were approved by the University Committee for Animal Welfare (UCAW) at the University of Louisville. All experiments were conducted in accordance with the approved UCAW protocol and applicable institutional and federal guidelines and regulations.

Female immunodeficient mice (NSG; 6–8 weeks old) were housed under specific pathogen–free conditions. Two estrogen receptor–positive (ER^+^) breast cancer patient-derived xenograft (PDX) models were used. TM00386 was obtained from The Jackson Laboratory PDX Resource and was derived from a primary invasive ER + ductal carcinoma of the breast. BCM15100 was obtained from the Baylor College of Medicine breast cancer PDX and advanced *in vivo* models core under a material transfer agreement and was derived from an ER^+^ infiltrating ductal carcinoma.

PDX tumor fragments (~ 2–3 mm^3^) were orthotopically implanted into the mammary fat pad. To support ER + tumor growth, mice received 17β-estradiol supplementation via drinking water (2.7 mg/mL; Sigma-Aldrich, Cat. No. E2758) for the duration of the study. Tumor volume was measured every two days using calipers and calculated as (length × width^2^)/2. Mice were randomized into treatment groups when tumors reached ~ 100–150 mm^3^. PFK-158·2HCl (Chemgood, Cat. No. PFK158) was formulated in 30% (w/v) Captisol (Selleckchem, Cat. No. S4592) and administered by intraperitoneal injection at a dose of 40 mg/kg. Palbociclib was formulated in sodium lactate and administered via gavage at a dose of 50 mg/kg. Vehicle-treated control mice received the corresponding formulation without active drug. Animals were monitored regularly for body weight, tumor burden, and overall health. In accordance with University of Louisville UCAW guidelines, the maximal permitted cumulative tumor burden was defined as a tumor diameter greater than 15 mm in an adult mouse, and this limit was not exceeded in any experiment. Animals were euthanized at predefined humane endpoints.

At the experimental endpoint, tumor volume was recorded, followed by nutrient tracing studies and euthanasia by cervical dislocation. Tumors were then excised and processed for downstream analyses, including mass spectrometry analysis and immunohistochemistry.

#### In Vivo [U-^13^C]-Glucose Infusion and Tumor Metabolite Analysis

At the experimental endpoint, tumor-bearing mice underwent *in vivo* glucose tracing studies to assess acute glucose utilization. Mice were injected via the tail vein with 80 μL of 25% (w/v) [U-^13^C]-glucose (Cambridge Isotope Laboratories, Cat. No. CLM-1396–1) per injection, administered three times at 15-minute intervals, as previously described(26). Fifteen minutes after the final injection, mice were euthanized by cervical dislocation in accordance with the approved UCAW protocol. Tumors were rapidly excised, weighed, and flash-frozen for downstream metabolomic analyses. For metabolite extraction, up to 15 mg of pulverized frozen tumor tissue was processed for analysis of polar and lipid metabolites. Metabolites were extracted using acetonitrile/H_2_O (60:40, v/v). Extracted metabolites were lyophilized and subsequently analyzed by LC/MS as described for the *in vitro* stable isotope tracing experiments.

### Immunohistochemistry

Tumor tissues were harvested at the experimental endpoint, fixed in 10% neutral-buffered formalin, and embedded in paraffin. Five-μm sections were cut, mounted on glass slides, deparaffinized in xylene, and rehydrated through graded ethanol to water. Antigen retrieval was performed using citrate buffer in a 2100 Retriever (PickCell Laboratories). Tissue sections were blocked with 10% goat serum for 1 hour and then incubated overnight at 4°C with primary antibodies. Proliferation was assessed using a Ki67 antibody (Abcam, ab16667) at a 1:50 dilution, and PFKFB3 expression was assessed using an anti-PFKFB3 antibody (Proteintech, 13763–1-AP) at a 1:100 dilution. Following primary antibody incubation, sections were washed and incubated with species-appropriate secondary antibodies. Staining was developed with 3,3′-diaminobenzidine tetrahydrochloride (DAB, Vector Laboratories, Burlingame, CA, USA), nuclei were counterstained with hematoxylin (Sigma-Aldrich), and coverslips attached (Permount, Fisher Scientific, Fairlawn, NJ, USA). Stained slides were digitally scanned using a 3DHistech PanDesk slide scanner (Epredia).

### Statistical analysis

Statistical analyses were carried out using GraphPad Prism (Version 10.6.1 for macOS, GraphPad Software, Boston, Massachusetts USA, www.graphpad.com). All numerical data are reported as mean ± S.D. Grouped analysis was performed using either two-way ANOVA or one-way ANOVA with corrections as indicated. Paired two-tailed Student’s t-tests were used for analyses involving matched or paired samples, as specified in the figure legends. For each experiment, replicates and p-values for all results are listed in their respective figure legends.

## Results

### CDK4/6 inhibition induces PFKFB3 and enhances glycolysis

**Figure 1.**
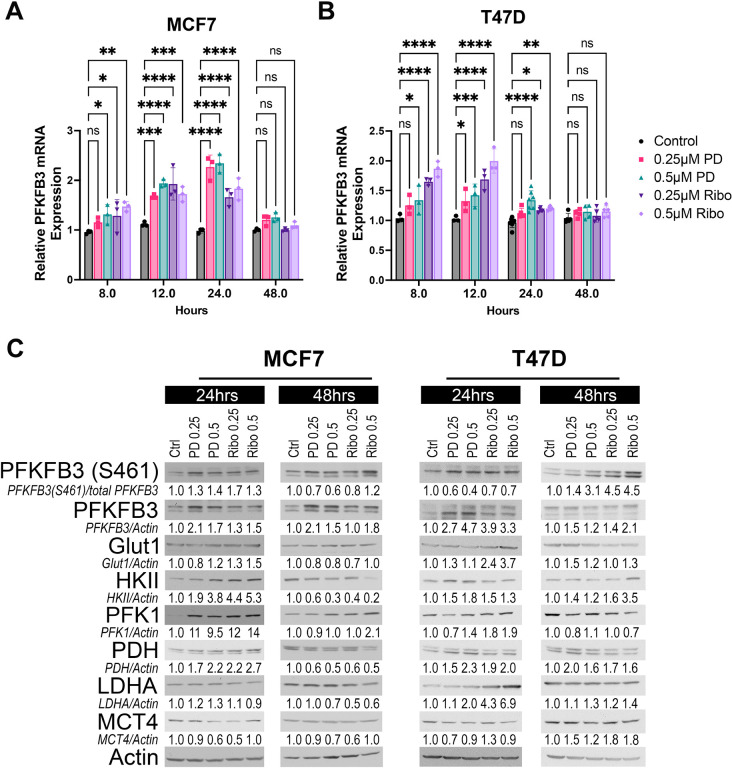
CDK4/6 inhibition induces PFKFB3 expression and phosphorylation in ER^+^ breast cancer cells. (A–B) Relative PFKFB3 mRNA expression in MCF7 (A) and T47D (B) cells treated with palbociclib (PD) or ribociclib (Ribo) at the indicated doses for 8–48 hours. Data are shown as mean ± SD of three independent experiments and analyzed by two-way ANOVA with multiple comparisons (*P < 0.05; **P < 0.01; ***P < 0.001; ****P < 0.0001; ns, not significant). (C) Representative immunoblots of PFKFB3 (total and Ser461-phosphorylated) and the indicated metabolic proteins in MCF7 and T47D cells treated with palbociclib 0.25 or 0.5 μM (PD) or ribociclib (Ribo) for 24 or 48 hours. Protein densitometry was performed using the Un-Scan-It software. Phospho-PFKFB3 (Ser461) was normalized to total PFKFB3, whereas all other proteins were normalized to β-actin. Quantified values are expressed relative to control.

To determine whether metabolic rewiring induced by CDK4/6 inhibition involves PFKFB3, we first examined PFKFB3 mRNA levels in ER^+^ MCF7 and T47D cells exposed to palbociclib and ribociclib for 8–48 hours. Both cell lines demonstrated temporal induction of PFKFB3, but with distinct patterns. In MCF7 cells, PFKFB3 mRNA increased rapidly following CDK4/6 inhibition, with significant induction as early as 8 hours and maximal expression at 12–24 hours prior to returning toward baseline by 48 hours (Fig. 1A). In contrast, T47D cells exhibited a more modest response, with peak PFKFB3 expression occurring between 8–12 hours and a greater magnitude of induction following ribociclib treatment (Fig. 1B). Since PFKFB3 mRNA increased following CDK4/6 inhibition, we next examined whether these transcriptional changes translated into increased protein levels. Endogenous PFKFB3 was markedly increased in both MCF7 and T47D cells after 24–48 hours of exposure to palbociclib or ribociclib (Fig. 1C). This increase was accompanied by enhanced phosphorylation of PFKFB3 at Ser461, indicating activation of its kinase function. In addition to PFKFB3, we observed a pronounced induction of PFK1, the downstream rate-limiting enzyme activated by PFKFB3-derived F2,6BP, with the highest increase seen in MCF7 cells where PFK1 increased 10–14-fold after 24 hours of treatment. Given that glycolytic flux is regulated at additional rate-limiting steps—including glucose uptake (GLUT1), glucose phosphorylation (HKII), and lactate export (MCT4), we next evaluated whether CDK4/6 inhibition altered the expression of these enzymes. GLUT1 expression changed only modestly across all treatment conditions. HKII showed a moderate but inconsistent increase, most evident in MCF7 cells at 24 hours but was not sustained at later timepoints. MCT4, which mediates lactate export and is often induced during high glycolytic activity, remained largely unchanged in both models. We also examined other glycolytic regulators, including LDHA and PDH, and found no consistent changes across treatments. Taken together, these findings indicate that CDK4/6 inhibition results in a selective and coordinated activation of the PFKFB3–PFK1 regulatory step, rather than a broad upregulation of the glycolytic machinery.

### PFKFB3 activation by CDK4/6 inhibition enhances glycolytic flux

**Figure 2.**
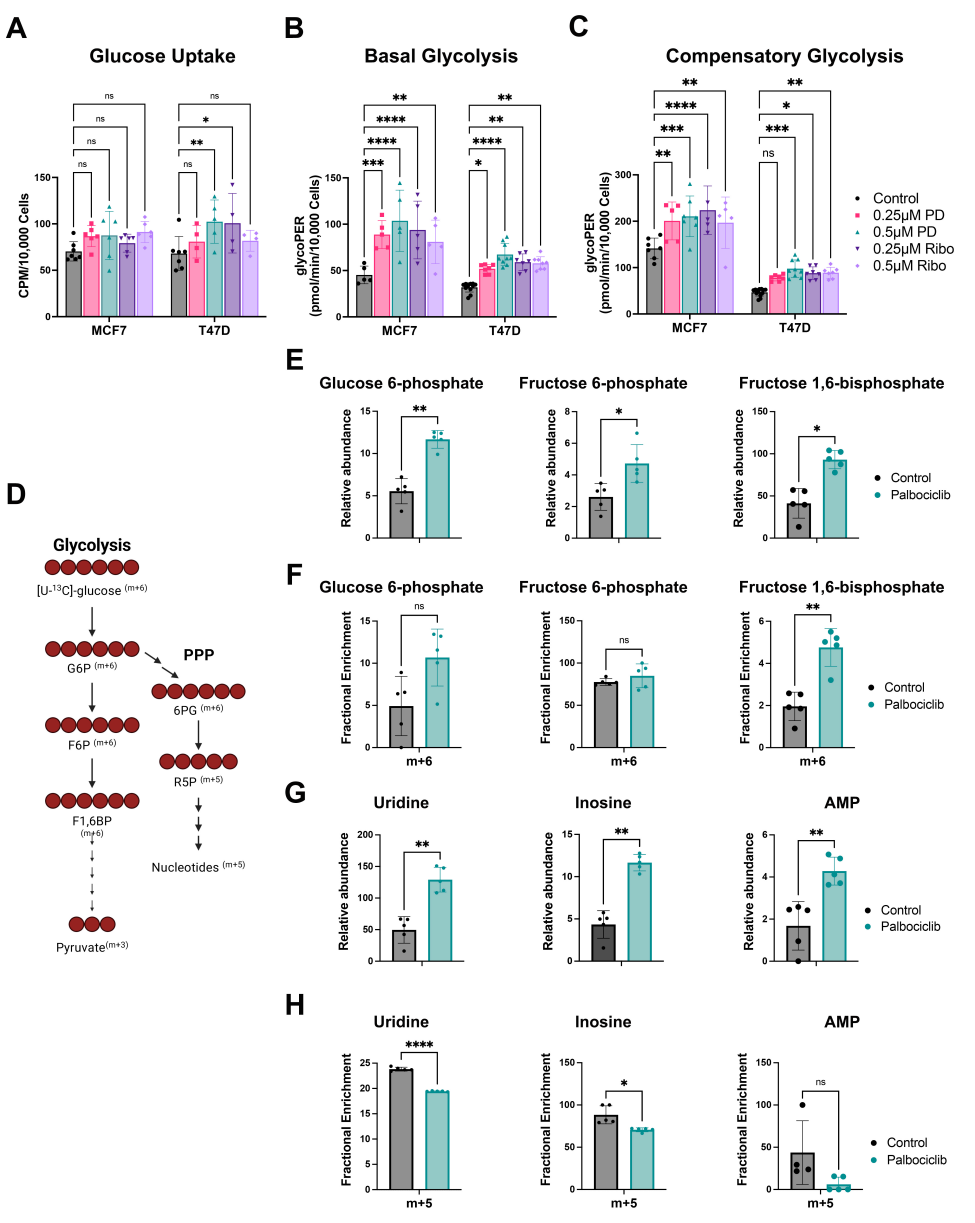
Glucose uptake, glycolytic flux, and metabolic analysis. (A) Glucose uptake in MCF7 and T47D cells treated with palbociclib (PD) or ribociclib (Ribo) at the indicated concentrations. Uptake is expressed as counts per minute (CPM) normalized to cell number. (B) Basal glycolysis and (C) compensatory glycolysis measured by Seahorse extracellular flux analysis following treatment with palbociclib or ribociclib. Glycolytic rates are expressed as proton efflux rate (PER) normalized to cell number. (D) Schematic of [U-^13^C]-glucose tracing, illustrating carbon flow through glycolysis and diversion into the pentose phosphate pathway (PPP) beginning at glucose-6-phosphate (G6P), leading to ribose-5-phosphate (R5P) and nucleotide synthesis. Expected mass isotopologues (m + 6 for hexose intermediates, m + 5 for ribose) are indicated. (E) Relative abundance of glycolytic intermediates (glucose-6-phosphate, fructose-6-phosphate, and fructose-1,6-bisphosphate) in MCF7 cells treated with control or 0.5μM palbociclib. (F) Fractional enrichment (m + 6) of glycolytic intermediates following [U-^13^C]-glucose tracing in MCF7 cells treated with control or 0.5μM palbociclib. (G) Relative abundance of nucleotide metabolites (uridine, inosine, and AMP) in MCF7 cells treated with control or 0.5μM palbociclib. (H) Fractional enrichment (m + 5) of nucleotide metabolites measured following [U-^13^C]-glucose tracing in MCF7 cells treated with control or 0.5μM palbociclib. Data are presented as mean ± SD of one experiment with 5 biological replicates. Two-way ANOVA with multiple-comparisons testing was used for panels A–C. Paired two-tailed Student’s t-tests were used for panels E–H. (*P < 0.05; **P < 0.01; ***P < 0.001; ****P < 0.0001; ns, not significant)

To determine whether increased PFKFB3 expression in response to CDK4/6 inhibition led to functional alterations in glucose catabolism, we next evaluated glucose uptake, glycolytic flux and metabolic fate of glucose following CDK4/6 inhibition. Glucose uptake was modestly increased in T47D cells but remained largely unchanged in MCF7 cells (Fig. 2A). In contrast, basal glycolysis was significantly elevated in both models following CDK4/6 inhibition (Fig. 2B), and this increase was accompanied by a marked rise in compensatory glycolysis, particularly in MCF7 cells (Fig. 2C), indicating enhanced glycolytic flux under CDK4/6 blockade.

To define how CDK4/6 inhibition alters glucose utilization, we first performed stable isotope tracing using [U-^13^C]-glucose, with metabolic pathways and isotopologue labeling patterns summarized in Fig. 2D. This schematic illustrates glucose flux through upper glycolysis to fructose 1,6-bisphosphate (F1,6BP; m + 6), with branch points directing glucose-derived intermediates toward the pentose phosphate pathway (PPP) and downstream nucleotide biosynthesis.

We initially quantified relative metabolite abundance in MCF7 cells following 48 hours of palbociclib exposure. Consistent with the activation of the PFKFB3-PFK1 regulatory step, CDK4/6 inhibition significantly increased the abundance of multiple glycolytic intermediates, including glucose 6-phosphate, fructose 6-phosphate, and fructose 1,6-bisphosphate (Fig. 2E). To determine whether these changes reflected increased carbon flux through glycolysis, we next examined isotopologue enrichment from [U-^13^C]-glucose. Although m + 6 fractional enrichment of glucose 6-phosphate and fructose 6-phosphate showed a modest upward trend, these differences were not statistically significant (Fig. 2F). Importantly, m + 6 fructose 1,6-bisphosphate was significantly increased, indicating enhanced glycolytic flux downstream of PFKFB3-PFK1.

We next examined pathways adjacent to glycolysis, focusing on the pentose phosphate pathway. Palbociclib treatment led to a marked increase in the relative abundance of nucleotide intermediates—including uridine, inosine and AMP (Fig, 2G). However, incorporation of ^13^C-glucose into the ribose moiety of these nucleotides (m + 5) was significantly reduced in the palbociclib treated samples (Fig. 2H). Given that m + 5 labeling derives from [U-^13^C]-glucose entering the oxidative PPP to generate m + 5 labeled ribose-5-phosphate, the discordance between increased abundance and decreased labeling indicates that nucleotide pools accumulate due to reduced utilization during G1 arrest rather than increased glucose-derived biosynthesis. Taken together, these findings demonstrate that CDK4/6 inhibition selectively enhances glycolytic flux through the PFKFB3-PFK1 node while simultaneously limiting glucose routing into nucleotide synthesis pathways.

### PFKFB3 knockdown impairs CDK4/6i-induced glycolytic activation

**Figure 3.**
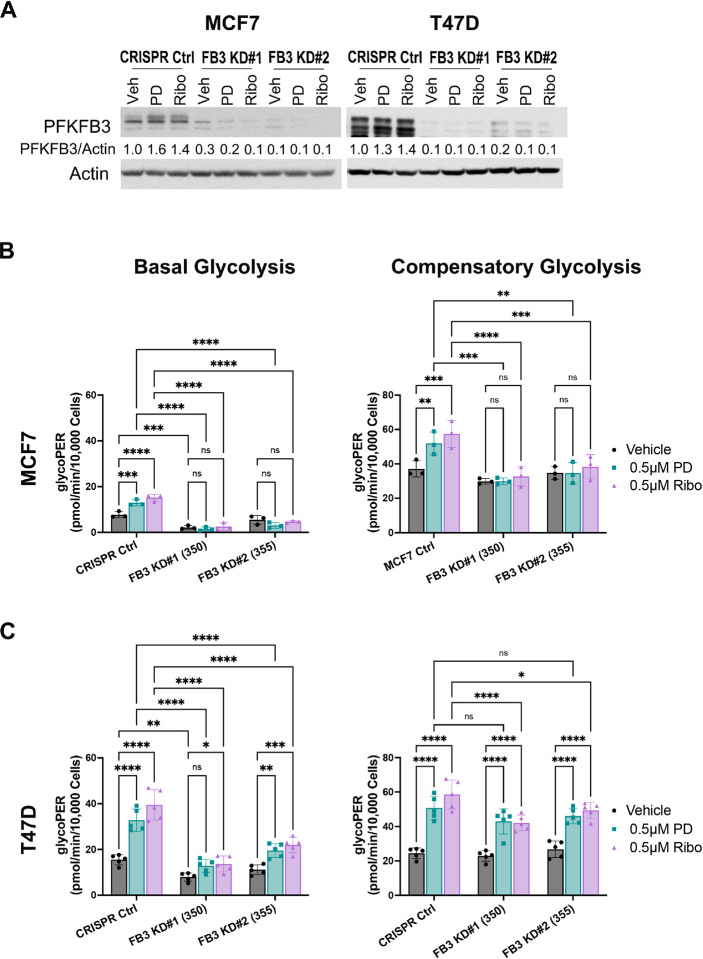
Requirement of PFKFB3 for CDK4/6 inhibitor–induced glycolytic responses. (A) Immunoblot analysis of PFKFB3 expression in MCF7 and T47D CRISPR control cells and two independent PFKFB3 knockdown (FB3 KD#1 and FB3 KD#2) cell lines treated with vehicle, 0.5μM palbociclib (PD), or 0.5μM ribociclib (Ribo). β-Actin was used as a loading control. Representative blots are shown with densitometric quantification normalized to β-actin and expressed relative to vehicle-treated CRISPR control cells. (B-C) Basal glycolysis and compensatory glycolysis were measured by Seahorse extracellular flux analysis in MCF7 (top) and T47D (bottom) CRISPR control and PFKFB3 knockdown cells following treatment with vehicle, 0.5 μM palbociclib (PD), or 0.5 μM ribociclib (Ribo). Glycolytic rates are expressed as proton efflux rate (PER) normalized to cell number. Data are presented as mean ± SD of three independent experiments, each performed with three biological replicates. Statistical analysis was performed using two-way ANOVA with multiple comparisons (*P < 0.05; **P < 0.01; ***P < 0.001; ****P < 0.0001; ns, not significant).

To determine whether PFKFB3 is required for the glycolytic reprogramming induced by CDK4/6 inhibition, we generated two independent CRISPR knockdown (KD) cell lines for PFKFB3 in MCF7 and T47D cells. Western blot analysis confirmed effective depletion of PFKFB3 protein, with loss of the palbociclib- and ribociclib-induced upregulation observed in CRISPR control cells (Fig. 3A).

Seahorse glycolytic rate assays revealed that in MCF7 cells, PFKFB3 deletion completely abrogated the increase in basal glycolysis induced by CDK4/6 inhibition (Fig. 3B, left). Basal glycolytic rates were also significantly reduced in vehicle-treated KD cells compared with CRISPR controls, indicating that PFKFB3 is required to maintain basal glycolytic flux in these cells. Compensatory glycolysis was fully suppressed in the KD lines following CDK4/6 treatment (Fig. 3B, right), demonstrating a critical dependence on PFKFB3 for both basal and drug-induced glycolytic capacity in MCF7 cells.

In T47D cells, PFKFB3 knockdown also significantly reduced basal and CDK4/6 inhibitor-induced glycolysis; however, the extent of suppression was less pronounced than in MCF7 cells (Fig. 3C, left). CDK4/6 inhibition still induced a modest but significant increase in basal glycolysis in KD cells, indicating that glycolytic activation is only partially attenuated in these cells by PFKFB3 abrogation. This incomplete suppression likely reflects the relatively high endogenous levels of PFKFB3 in T47D cells and/or compensatory engagement of additional glycolytic regulators. Similarly, compensatory glycolysis in CDK4/6 inhibitor-treated was significantly blocked in PFKFB3-deficient T47D cells but not fully abolished (Fig. 3C, right). Overall, these data support a central role for PFKFB3 in mediating the adaptive glycolytic response induced by CDK4/6 inhibition.

### PFKFB3 inhibition synergizes with CDK4/6 blockade in ER + breast cancer cells

**Figure 4.**
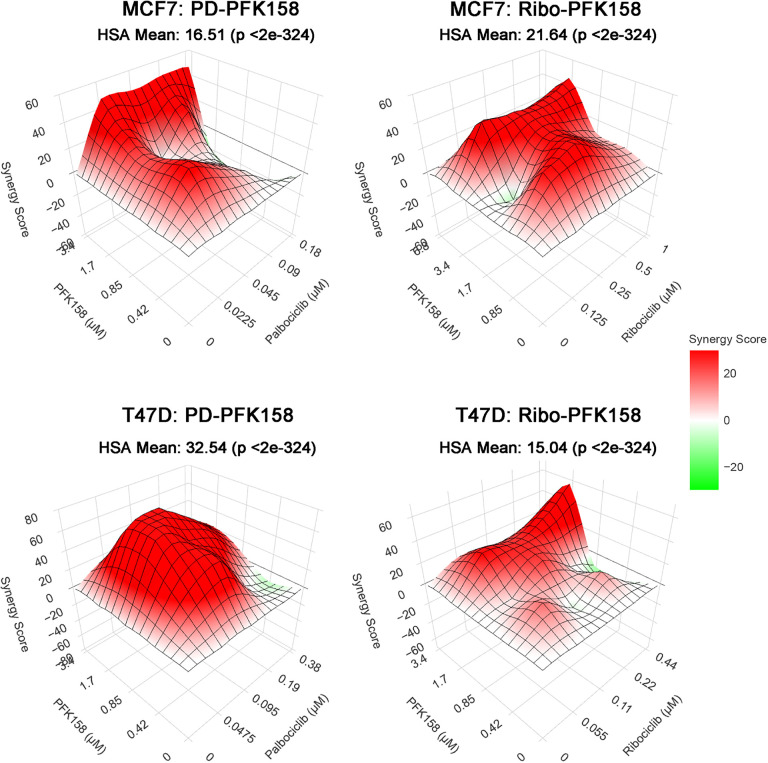
Synergy analysis of CDK4/6 inhibitors combined with PFKFB3 inhibition in ER^+^ breast cancer cells. MCF7 (top) and T47D (bottom) cells were treated with increasing concentrations of CDK4/6 inhibitors in combination with the PFKFB3 inhibitor PFK-158 across the indicated dose matrices. Drug combination response values were used to calculate synergy scores using the SynergyFinder web application with the Highest Single Agent (HSA) model. Three-dimensional response surface plots depict HSA synergy scores across drug combinations, with positive values indicating synergy and negative values indicating antagonism. Mean HSA synergy scores with associated *p*-values for each condition are shown in each plot. Data represent at least three independent experiments, each performed with four biological replicates.

Given our findings that CDK4/6 inhibition upregulates glycolysis in a PFKFB3-dependent manner, we hypothesized that dual inhibition of CDK4/6 and PFKFB3 would result in a synergistic anti-proliferative effect. To test this, we performed drug synergy assays in MCF7 and T47D ER + breast cancer cell lines using a dosing matrix of palbociclib or ribociclib in combination with the PFKFB3 inhibitor PFK-158. Antiproliferative synergy was quantified using the Highest Single Agent (HSA) model, with 3D response surfaces illustrating synergy scores across drug concentration matrices (Fig. 4). Positive scores (red) indicate synergy, while negative scores (green) reflect antagonism. In MCF7 cells, both palbociclib-PFK-158 and ribociclib-PFK-158 combinations demonstrated moderate synergy, with HSA mean scores of 16.51 and 21.64, respectively. In T47D cells, synergy was more pronounced; palbociclib-PFK-158 combination produced a strong synergistic interaction (HSA mean = 32.54), while ribociclib-PFK-158 also showed significant synergy, though to a lesser extent (HSA mean = 15.04). These results demonstrate that combined inhibition of CDK4/6 and PFKFB3 is more effective than either drug alone and support a therapeutic strategy targeting both cell cycle progression and glycolytic adaptation in ER + breast cancer.

### Dual CDK4/6 and PFKFB3 inhibition enhances antitumor activity in ER + PDX models

**Figure 5.**
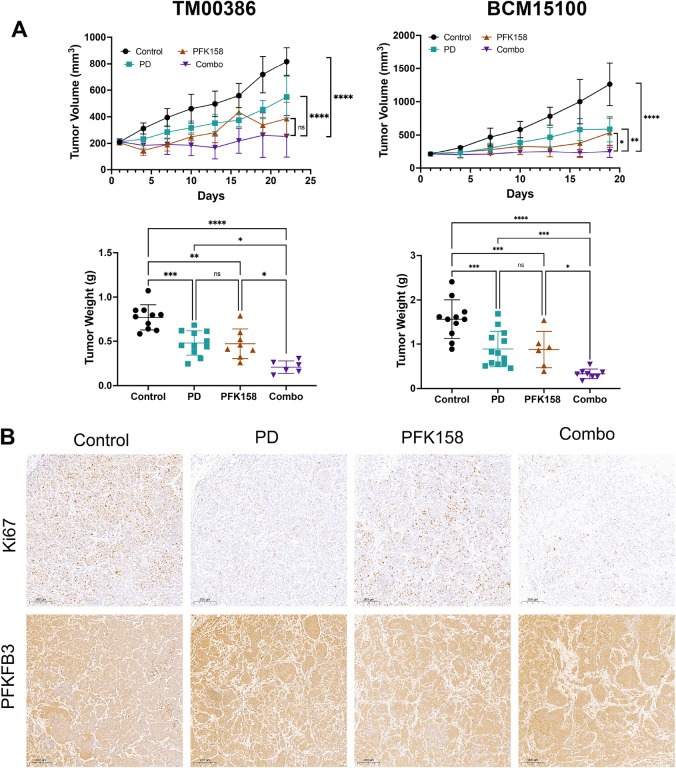
Combined CDK4/6 and PFKFB3 inhibition reduces tumor growth in ER^+^ PDX breast cancer models. (A) Tumor growth curves (top) and tumor weights at endpoint (bottom) from ER^+^ PDX models TM00386 (left) and BCM15100 (right). Mice were treated with vehicle control, palbociclib (50 mg/kg), PFK-158 (40 mg/kg), or the combination. Tumor volume was measured over the indicated time course, and tumor weights were recorded at the study endpoint. Data are presented as mean ± SD for tumor volume and as individual values with mean ± SD for tumor weight. Statistical analysis was performed using two-way ANOVA with multiple comparisons testing. (B) Representative immunohistochemical staining of tumor sections from PDX models treated with vehicle control, palbociclib, PFK-158, or the combination. Sections were stained for Ki67 and PFKFB3. Images shown are representative of tumors from BCM15100-treated animals.

We evaluated the therapeutic efficacy of combined CDK4/6 and PFKFB3 inhibition *in vivo* using two patient-derived xenograft (PDX) models of ER + breast cancer. Mice bearing TM00386 or BCM15100 tumors were treated with vehicle, palbociclib (50mg/kg), PFK-158 (40mg/kg), or their combination. In both PDX models, palbociclib and PFK-158 monotherapies exerted moderate tumor growth suppression; however, the combination produced the most pronounced antitumor effect, resulting in marked reduction of tumor volumes over the 21-day treatment period compared with either single agent (Fig. 5A). Consistent with these longitudinal tumor volume measurements, endpoint tumor weights were significantly decreased in the combination-treated animals in both PDX models (Fig. 5A). To assess treatment-associated changes in proliferation, we performed immunohistochemical analysis of Ki67. Combination-treated tumors showed the lowest Ki67 staining, indicating reduced proliferative activity compared to monotherapy or control tumors (Fig. 5B; representative images shown for BCM15100). PFKFB3 expression remained readily detectable across all treatment groups, consistent with the pharmacological mode of action of PFK-158. Notably, qualitative assessment of PFKFB3 staining suggested a trend toward increased expression in tumors treated with palbociclib alone and in combination, consistent with our *in vitro* findings demonstrating upregulation of PFKFB3 following CDK4/6 inhibition. Together, these findings indicate that pharmacological inhibition of PFKFB3 significantly enhances the antitumor activity of CDK4/6 inhibition *in vivo*—despite preserved or potentially increased—PFKFB3 levels.

#### CDK4/6 and PFKFB3 inhibition restricts glucose-derived carbon utilization in vivo

**Figure 6.**
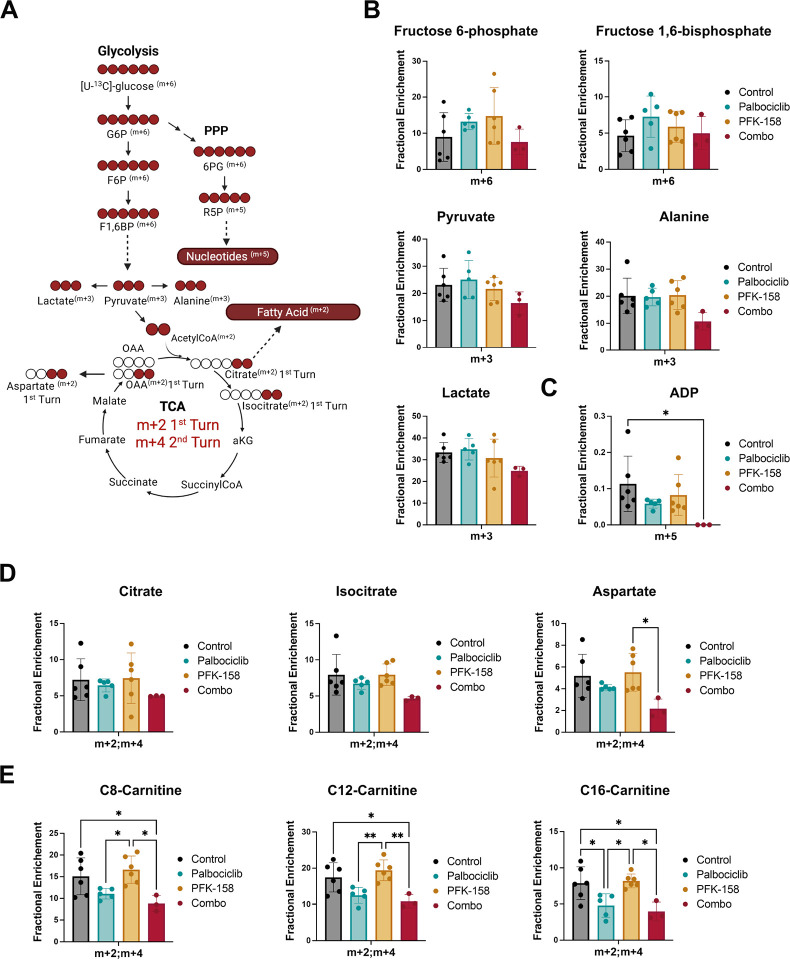
Metabolic analyses following combined CDK4/6 and PFKFB3 inhibition in vivo. TM00386 PDX-bearing mice were treated with vehicle control, 50mg/kg palbociclib, 40mg/kg PFK-158, or the combination, and subjected to *in vivo* [U-^13^C]-glucose bolus administration at endpoint, as described in Methods. (A) Schematic of [U-^13^C]-glucose tracing, illustrating carbon flow through glycolysis, diversion into the PPP, entry into the TCA cycle, and incorporation into amino acids, nucleotides, and fatty acid–associated metabolites. Expected isotopologues are indicated (m + 6 for hexose intermediates, m + 3 for pyruvate-derived metabolites, m + 5 for ribose-containing metabolites, and m + 2/m + 4 for metabolites derived from the first and second TCA cycle turns). (B) Fractional enrichment of glycolytic intermediates (fructose 6-phosphate, fructose 1,6-bisphosphate, pyruvate, alanine, and lactate). (C) Fractional enrichment (m + 5) of ADP. (D) Fractional enrichment of TCA cycle–related metabolites (citrate, isocitrate, and aspartate; m + 2/m + 4). (E) Fractional enrichment of acyl-carnitine species (C8-, C12-, and C16-carnitine; m + 2/m + 4). Data are presented as mean ± SD. Statistical analysis was performed using one-way ANOVA with Tukey’s post-hoc comparison.

To determine how combined CDK4/6 and PFKFB3 inhibition alter glucose metabolism *in vivo*, we performed [U-^13^C]-glucose tracing at the experimental endpoint and quantified tumoral fractional enrichment across major metabolic pathways. The schematic in Fig. 6A summarizes the metabolic routes interrogated, illustrating glucose flux through glycolysis and its branching into the PPP, the tricarboxylic acid (TCA) cycle, and fatty acid biosynthesis.

We first assessed glucose-derived carbon incorporation into glycolytic intermediates as illustrated in Fig. 6A. Consistent with our *in vitro* findings, early glycolytic intermediates displayed detectable incorporation of ^13^C glucose-derived in all treatment groups (Fig. 6B). Although not statistically significant, fractional ^13^C enrichment of fructose 6-phosphate, fructose 1,6-bisphosphate, pyruvate, alanine, and lactate was reduced in the combination treated groups compared to each monotherapy.

Next, we analyzed glucose entry into anabolic pathways, focusing on ribose labeling of ADP as a readout for PPP activity (Fig. 6C). Palbociclib treatment significantly reduced m + 5 incorporation into ADP, and ^13^C-labeled ADP was undetectable in the combination group. These findings indicate that CDK4/6 inhibition suppresses nucleotide synthesis *in vivo*, with further loss of PPP-derived ribose production with the addition of the PFKFB3 inhibitor.

To determine whether glucose-derived carbon was incorporated into mitochondrial metabolism, we analyzed labeling of TCA cycle intermediates (Fig. 6D). Fractional enrichment of citrate and isocitrate (m + 2/m + 4), reflecting entry of labeled acetyl-CoA via pyruvate dehydrogenase, was moderately decreased in combination-treated tumors. Aspartate, which reflects further TCA labeling via oxaloacetate (OAA), showed a significant reduction in fractional enrichment with combination treatment. These results demonstrate that dual palbociclib and PFK-158 inhibition limits mitochondrial oxidation of glucose-derived carbons *in vivo*.

Lastly, we examined fatty acid labeling using short (C8)-, medium (C12)-, and long (C16)-chain acyl-carnitine labeling as a surrogate for incorporation of glucose-derived acetyl-CoA into fatty acids (Fig. 6E). Palbociclib significantly reduced the m + 2/m + 4 labeling of C8-, C12-, C16-carnitines and this effect was modestly amplified by the combination treatment but not statistically significant. These findings indicate a marked block of glucose incorporation into fatty acids *in vivo* driven by palbociclib.

Taken together, these *in vivo* tracing studies demonstrate that combined CDK4/6 and PFKFB3 inhibition broadly restricts the routing of glucose-derived carbons into nucleotides, mitochondrial metabolism, and fatty acids. This coordinated disruption of glucose-dependent anabolic and mitochondrial metabolism provides a mechanistic basis for the enhanced antitumor activity observed with dual CDK4/6 and PFKFB3 inhibition.

## Discussion

While cancer metabolism has long been considered a therapeutic vulnerability, the metabolic adaptations that emerge in response to targeted therapies, in particular CDK4/6 inhibitors, remain incompletely defined(27, 28). Emerging evidence indicates that CDK4/6 signaling intersects with metabolic regulation through both RB-dependent transcriptional programs and direct effects on metabolic enzymes; however, how these changes reshape glucose utilization in tumors has not been fully elucidated(14, 29, 30). Given the widespread adoption of CDK4/6 inhibitors as first-line therapy in combination with antiestrogens in ER + breast cancer patients, a better understanding of their metabolic consequences is warranted.

In this study, we demonstrate that CDK4/6 inhibition induces a distinct metabolic state in ER + breast cancer cells characterized by increased glycolytic flux through the PFKFB3-PFK1 regulatory step coupled with reduced routing of glucose-derived carbon into downstream biosynthetic pathways. Notably, this metabolic phenotype was observed across multiple clinically approved CDK4/6 inhibitors, despite their known differences in kinase selectivity and pharmacologic properties, indicating that rewiring of glycolytic regulation represents a conserved consequence of CDK4/6 pathway inhibition(31–33). Palbociclib treatment increased glycolytic activity, as evidenced by accumulation of early glycolytic intermediates and enhanced labeling of fructose 1,6-bisphosphate. Notably, despite this increase in glycolytic flux, incorporation of glucose-derived carbons into nucleotide synthesis and fatty acid biosynthesis was markedly reduced. These findings indicate that CDK4/6 inhibition uncouples glycolytic flux from anabolic glucose utilization, consistent with reduced biosynthetic demand during G1 cell cycle arrest.

PFKFB3 has been extensively implicated in sustaining high glycolytic flux in breast cancer and other solid tumors, positioning it as a key regulatory node linking proliferative signaling to glucose metabolism(20, 22, 24, 34). In our study, CDK4/6 inhibition consistently increased basal and compensatory glycolysis, and genetic silencing of PFKFB3 abrogated the CDK4/6 inhibitor-induced increase in glycolytic flux. These findings differ from reports by Lorito *et al*., who observed reduced glycolytic capacity upon CDK4/6 inhibition(19). A likely explanation for this apparent contradiction lies in differences in normalization strategies. Normalization-dependent artifacts have been described previously in settings where cell size, protein content, or mitochondrial mass are altered(23). In prior studies, extracellular acidification rates were normalized to protein content, an approach that can be confounded when cell cycle arrest is accompanied by continued biomass accumulation. CDK4/6 inhibitor-treated cells undergo G1 arrest while continuing to accumulate biomass and protein content, rendering normalization to protein content or mitochondrial markers susceptible to underestimation of glycolytic activity. In contrast, normalization to cell number reveals an increase in glycolytic flux in response to CDK4/6 inhibition. This approach reconciles discrepancies with previous reports and supports the conclusion that CDK4/6 inhibition enhances glycolysis.

Our *in vitro* metabolomics data further support the increase in glycolysis in response to CDK4/6 inhibition as shown by the accumulation of glycolytic intermediates and increased carbon incorporation into fructose 1,6-bisphosphate. *In vivo* tracing studies revealed more modest changes in glycolytic intermediates, which is not unexpected given the complexity of the tumor microenvironment and the absence of fasting prior to tracer administration. Moreover, tumor bulk measurements incorporate stromal, immune and endothelial cells that raise the potential for diluting tumor-specific metabolic changes. Despite these confounding factors, directional increases in upstream glycolytic intermediates were preserved *in vivo*, supporting our conclusion that glycolysis is activated under CDK4/6 inhibition.

In contrast, the most pronounced metabolic effects observed *in vivo* occurred downstream of glycolysis, with reduced incorporation of glucose-derived carbon into lipid-associated metabolites and nucleotides. These changes are consistent with reduced demand for *de novo* lipid synthesis and nucleotide production during CDK4/6-induced cell cycle arrest. Proliferating tumor cells require sustained anabolic flux to support membrane biogenesis and DNA replication; thus, inhibition of proliferation would be expected to limit glucose routing into lipogenic and nucleotide biosynthetic pathways. Consistent with this interpretation, both *in vitro* and *in vivo* metabolomic analyses demonstrated reduced glucose contribution to nucleotide pools. Prior studies have shown that palbociclib suppresses PPP activity through inhibition of glucose 6-phosphate dehydrogenase, resulting in diminished ribose 5-phosphate production and impaired nucleotide synthesis(16). Although these studies were performed in a different tumor type, they provide independent support for the finding that CDK4/6 inhibition limits glucose entry into nucleotide biosynthesis. Together, these findings support a model in which reduced nucleotide labeling reflects decreased anabolic demand reinforced by restricted pentose phosphate pathway flux, rather than impaired glucose uptake or glycolytic capacity. A limitation of this study is that bulk metabolomics analysis does not resolve cell type-specific metabolic responses within the tumor microenvironment; future studies incorporating special or single cell approaches will be valuable to further refine these findings.

Importantly, despite restricted downstream anabolic glucose utilization, glycolytic activity itself remains elevated under CDK4/6 inhibition and likely serves functions beyond biomass production, including maintenance of ATP levels, redox balance, and metabolic homeostasis in G1-arrested cells. In this context, glycolysis appears to support tumor cell maintenance rather than proliferation. Disruption of this adaptive metabolic state through PFKFB3 inhibition limits glucose utilization and compromises the ability of CDK4/6-inhibited cells to maintain metabolic balance.

Consistent with this framework, co-targeting PFKFB3 further restricted glucose utilization across multiple downstream pathways and enhanced the antitumor efficacy of CDK4/6 inhibition *in vivo*. Together, these findings support a model in which CDK4/6 inhibition rewires metabolism to reduce biosynthetic demand while simultaneously increasing reliance on regulated glycolytic flux for metabolic homeostasis. Targeting PFKFB3 exploits this adaptive metabolic vulnerability and provides a mechanistic rationale for combining glycolytic inhibition with CDK4/6-targeted therapy in ER + breast cancer.

## Conclusions

Our findings demonstrate that CDK4/6 inhibition rewires glucose metabolism in ER^+^ breast cancer by increasing glycolytic flux while limiting downstream anabolic utilization. Targeting this adaptive metabolic state through PFKFB3 inhibition enhances the antitumor efficacy of CDK4/6 inhibitors and supports the therapeutic potential of combining glycolytic modulation with CDK4/6-directed therapy.

## Supplementary Material

Supplementary Files

This is a list of supplementary files associated with this preprint. Click to download.

- WesternBlotsFigure1originalscansT47DCELLS.pdf
- WesternBlotsFigure1originalscansMCF7CELLS.pdf
- WesternBlotsFigure3originalscansFN.pdf

## Data Availability

All data generated or analyzed during this study are included in this published article.

## Abbreviations

ADP: Adenosine diphosphate
AI: Aromatase inhibitor
ANOVA: Analysis of variance
AMP: Adenosine monophosphate
ATP: Adenosine triphosphate
Cas9: CRISPR–associated protein 9
CDK4/6: Cyclin–dependent kinase 4 and 6
CRISPR: Clustered regularly interspaced short palindromic repeats
ECAR: Extracellular acidification rate
ER / ER^+^: Estrogen receptor / estrogen receptor–positive
ERα: Estrogen receptor alpha
F2,6BP: Fructose–2,6–bisphosphate
F6P: Fructose–6–phosphate
G1: Gap 1 phase of the cell cycle
G6P: Glucose–6–phosphate
GLUT1: Glucose transporter 1
HER2: Human epidermal growth factor receptor 2
HK2: Hexokinase II
LC/MS: Liquid chromatography–mass spectrometry
LDHA: Lactate dehydrogenase A
OAA: Oxaloacetate
PDH: Pyruvate dehydrogenase
PDX: Patient–derived xenograft
PFK: 1–Phosphofructokinase–1
PFKFB3: 6–Phosphofructo–2–kinase/fructose–2,6–bisphosphatase 3
pPFKFB3 (Ser461): Phosphorylated PFKFB3 at serine 461
PPP: Pentose phosphate pathway
R5P: Ribose–5–phosphate
Rb: Retinoblastoma protein
Ribo: Ribociclib
SD: Standard deviation
TCA: Tricarboxylic acid (cycle)
^13^C: Carbon–13 isotope

## References

[1] Ali S, Coombes RC. Endocrine-responsive breast cancer and strategies for combating resistance. Nat Rev Cancer. 2002;2(2):101–12. 12635173 10.1038/nrc721

[2] Kim N, Lukong KE. Treating ER-positive breast cancer: a review of the current FDA-approved SERMs and SERDs and their mechanisms of action. Oncol Reviews. 2025;Volume 19–2025. 10.3389/or.2025.1564642 PMC12018393 40275985

[3] Gnant M, Turner NC, Hernando C. Managing a Long and Winding Road: Estrogen Receptor–Positive Breast Cancer. Am Soc Clin Oncol Educational Book. 2023(43):e390922. 37319380 10.1200/EDBK_390922

[4] Bidard FC, Kaklamani VG, Neven P, Streich G, Montero AJ, Forget F, Elacestrant (oral selective estrogen receptor degrader) Versus Standard Endocrine Therapy for Estrogen Receptor-Positive, Human Epidermal Growth Factor Receptor 2-Negative Advanced Breast Cancer: Results From the Randomized Phase III EMERALD Trial. J Clin Oncol. 2022;40(28):3246–56. 35584336 10.1200/JCO.22.00338 PMC9553388

[5] Burstein HJ, Somerfield MR, Barton DL, Dorris A, Fallowfield LJ, Jain D, Endocrine Treatment and Targeted Therapy for Hormone Receptor–Positive, Human Epidermal Growth Factor Receptor 2–Negative Metastatic Breast Cancer: ASCO Guideline Update. J Clin Oncol. 2021;39(35):3959–77. 34324367 10.1200/JCO.21.01392 PMC8659999

[6] Finn RS, Crown JP, Lang I, Boer K, Bondarenko IM, Kulyk SO, The cyclin-dependent kinase 4/6 inhibitor palbociclib in combination with letrozole versus letrozole alone as first-line treatment of oestrogen receptor-positive, HER2-negative, advanced breast cancer (PALOMA-1/TRIO-18): a randomised phase 2 study. Lancet Oncol. 2015;16(1):25–35. 25524798 10.1016/S1470-2045(14)71159-3

[7] Hortobagyi GN, Stemmer SM, Burris HA, Yap YS, Sonke GS, Paluch-Shimon S, Ribociclib as First-Line Therapy for HR-Positive, Advanced Breast Cancer. N Engl J Med. 2016;375(18):1738–48. 27717303 10.1056/NEJMoa1609709

[8] Hortobagyi GN, Stemmer SM, Burris HA, Yap YS, Sonke GS, Hart L, Overall Survival with Ribociclib plus Letrozole in Advanced Breast Cancer. N Engl J Med. 2022;386(10):942–50. 35263519 10.1056/NEJMoa2114663

[9] Sledge GW Jr., Toi M, Neven P, Sohn J, Inoue K, Pivot X, The Effect of Abemaciclib Plus Fulvestrant on Overall Survival in Hormone Receptor-Positive, ERBB2-Negative Breast Cancer That Progressed on Endocrine Therapy-MONARCH 2: A Randomized Clinical Trial. JAMA Oncol. 2020;6(1):116–24. 31563959 10.1001/jamaoncol.2019.4782 PMC6777264

[10] Li J, Huo X, Zhao F, Ren D, Ahmad R, Yuan X, Association of Cyclin-Dependent Kinases 4 and 6 Inhibitors With Survival in Patients With Hormone Receptor–Positive Metastatic Breast Cancer: A Systematic Review and Meta-analysis. JAMA Netw Open. 2020;3(10):e2020312–e. 33048129 10.1001/jamanetworkopen.2020.20312 PMC8094425

[11] Finn RS, Dering J, Conklin D, Kalous O, Cohen DJ, Desai AJ, PD 0332991, a selective cyclin D kinase 4/6 inhibitor, preferentially inhibits proliferation of luminal estrogen receptor-positive human breast cancer cell lines in vitro. Breast Cancer Res. 2009;11(5):R77. 19874578 10.1186/bcr2419 PMC2790859

[12] Watt AC, Goel S. Cellular mechanisms underlying response and resistance to CDK4/6 inhibitors in the treatment of hormone receptor-positive breast cancer. Breast Cancer Res. 2022;24(1):17. 35248122 10.1186/s13058-022-01510-6 PMC8898415

[13] Zhang B, Li D, Jin X, Zhang K. The CDK4/6 inhibitor PD0332991 stabilizes FBP1 by repressing MAGED1 expression in pancreatic ductal adenocarcinoma. Int J Biochem Cell Biol. 2020;128:105859. 32987196 10.1016/j.biocel.2020.105859

[14] Cretella D, Ravelli A, Fumarola C, La Monica S, Digiacomo G, Cavazzoni A, The anti-tumor efficacy of CDK4/6 inhibition is enhanced by the combination with PI3K/AKT/mTOR inhibitors through impairment of glucose metabolism in TNBC cells. J Exp Clin Cancer Res. 2018;37(1):72. 29587820 10.1186/s13046-018-0741-3 PMC5872523

[15] Tarrado-Castellarnau M, de Atauri P, Tarrago-Celada J, Perarnau J, Yuneva M, Thomson TM, De novo MYC addiction as an adaptive response of cancer cells to CDK4/6 inhibition. Mol Syst Biol. 2017;13(10):940. 28978620 10.15252/msb.20167321 PMC5658703

[16] Conroy LR, Lorkiewicz P, He L, Yin X, Zhang X, Rai SN, Palbociclib treatment alters nucleotide biosynthesis and glutamine dependency in A549 cells. Cancer Cell Int. 2020;20:280. 32624705 10.1186/s12935-020-01357-x PMC7329430

[17] Santiappillai NT, Abuhammad S, Slater A, Kirby L, McArthur GA, Sheppard KE CDK4/6 Inhibition Reprograms Mitochondrial Metabolism in BRAF(V600) Melanoma via a p53 Dependent Pathway. Cancers (Basel). 2021;13(3). 10.3390/cancers13030524 PMC7866416 33572972

[18] Franco J, Balaji U, Freinkman E, Witkiewicz AK, Knudsen ES. Metabolic Reprogramming of Pancreatic Cancer Mediated by CDK4/6 Inhibition Elicits Unique Vulnerabilities. Cell Rep. 2016;14(5):979–90. 26804906 10.1016/j.celrep.2015.12.094 PMC4757440

[19] Lorito N, Bacci M, Smiriglia A, Mannelli M, Parri M, Comito G Glucose Metabolic Reprogramming of ER Breast Cancer in Acquired Resistance to the CDK4/6 Inhibitor Palbociclib(). Cells. 2020;9(3). 10.3390/cells9030668 PMC7140692 32164162

[20] Yalcin A, Telang S, Clem B, Chesney J. Regulation of glucose metabolism by 6-phosphofructo-2-kinase/fructose-2,6-bisphosphatases in cancer. Exp Mol Pathol. 2009;86(3):174–9. 19454274 10.1016/j.yexmp.2009.01.003

[21] Jones BC, Pohlmann PR, Clarke R, Sengupta S. Treatment against glucose-dependent cancers through metabolic PFKFB3 targeting of glycolytic flux. Cancer Metastasis Rev. 2022;41(2):447–58. 35419769 10.1007/s10555-022-10027-5

[22] Imbert-Fernandez Y, Clem BF, O’Neal J, Kerr DA, Spaulding R, Lanceta L, Estradiol stimulates glucose metabolism via 6-phosphofructo-2-kinase (PFKFB3). J Biol Chem. 2014;289(13):9440–8. 24515104 10.1074/jbc.M113.529990 PMC3979387

[23] Foy R, Lew KX, Saurin AT. The search for CDK4/6 inhibitor biomarkers has been hampered by inappropriate proliferation assays. npj Breast Cancer. 2024;10(1):19. 38438376 10.1038/s41523-024-00624-8 PMC10912267

[24] Lypova N, Dougherty SM, Clem BF, Feng J, Yin X, Zhang X, PFKFB3-dependent redox homeostasis and DNA repair support cell survival under EGFR-TKIs in non-small cell lung carcinoma. Cancer Metab. 2024;12(1):37. 39696407 10.1186/s40170-024-00366-y PMC11658331

[25] Zheng S, Wang W, Aldahdooh J, Malyutina A, Shadbahr T, Tanoli Z, SynergyFinder Plus: Toward Better Interpretation and Annotation of Drug Combination Screening Datasets. Genom Proteom Bioinform. 2022;20(3):587–96. 10.1016/j.gpb.2022.01.004 PMC9801064 35085776

[26] Conroy LR, Dougherty S, Kruer T, Metcalf S, Lorkiewicz P, He L Loss of Rb1 Enhances Glycolytic Metabolism in Kras-Driven Lung Tumors In Vivo. Cancers (Basel). 2020;12(1). 10.3390/cancers12010237 PMC7016860 31963621

[27] Stine ZE, Schug ZT, Salvino JM, Dang CV. Targeting cancer metabolism in the era of precision oncology. Nat Rev Drug Discov. 2022;21(2):141–62. 34862480 10.1038/s41573-021-00339-6 PMC8641543

[28] DeBerardinis RJ, Chandel NS. Fundamentals of cancer metabolism. Sci Adv. 2016;2(5):e1600200. 27386546 10.1126/sciadv.1600200 PMC4928883

[29] Wu H, Kren BT, Lane AN, Cassel TA, Higashi RM, Fan TWM, Cyclin D1 extensively reprograms metabolism to support biosynthetic pathways in hepatocytes. J Biol Chem. 2023;299(12):105407. 38152849 10.1016/j.jbc.2023.105407 PMC10687208

[30] Wang H, Nicolay BN, Chick JM, Gao X, Geng Y, Ren H, The metabolic function of cyclin D3-CDK6 kinase in cancer cell survival. Nature. 2017;546(7658):426–30. 28607489 10.1038/nature22797 PMC5516959

[31] George MA, Qureshi S, Omene C, Toppmeyer DL, Ganesan S. Clinical and Pharmacologic Differences of CDK4/6 Inhibitors in Breast Cancer. Front Oncol. 2021;11:693104. 34327137 10.3389/fonc.2021.693104 PMC8313476

[32] Wang X, Zhao S, Xin Q, Zhang Y, Wang K, Li M. Recent progress of CDK4/6 inhibitors’ current practice in breast cancer. Cancer Gene Ther. 2024;31(9):1283–91. 38409585 10.1038/s41417-024-00747-x PMC11405274

[33] Grinshpun A, Tolaney SM, Burstein HJ, Jeselsohn R, Mayer EL. The dilemma of selecting a first line CDK4/6 inhibitor for hormone receptor-positive/HER2-negative metastatic breast cancer. npj Breast Cancer. 2023;9(1):15. 36949066 10.1038/s41523-023-00520-7 PMC10033931

[34] O’Neal J, Clem A, Reynolds L, Dougherty S, Imbert-Fernandez Y, Telang S, Inhibition of 6-phosphofructo-2-kinase (PFKFB3) suppresses glucose metabolism and the growth of HER2 + breast cancer. Breast Cancer Res Treat. 2016;160(1):29–40. 27613609 10.1007/s10549-016-3968-8

